# Cancer-Associated Abdominal Vein Thrombosis

**DOI:** 10.3390/cancers15215293

**Published:** 2023-11-04

**Authors:** Lorna Muscat-Baron, Amber Leigh Borg, Laura Maria Attard, Alex Gatt, Nicoletta Riva

**Affiliations:** 1 Medical School, Faculty of Medicine and Surgery, University of Malta, MSD 2080 Msida, Malta; lorna.muscat.17@um.edu.mt (L.M.-B.); amber-leigh.borg.18@um.edu.mt (A.L.B.); laura.m.attard.16@um.edu.mt (L.M.A.); 2 Department of Pathology, Faculty of Medicine and Surgery, University of Malta, MSD 2080 Msida, Malta; alexander.gatt@um.edu.mt

**Keywords:** cancer, venous thromboembolism, splanchnic circulation, ovary, renal veins

## Abstract

**Simple Summary:**

Cancer is associated with a high risk of developing venous thromboembolism, which includes thrombosis in unusual areas such as the abdominal veins (splanchnic, ovarian and renal veins). These thromboses are often incidental findings in the workup of a cancer patient. Cancer is one of the major risk factors for splanchnic vein thrombosis, ovarian vein thrombosis and renal vein thrombosis. Cancer-associated abdominal vein thrombosis carries high mortality rates and high risk of recurrent thrombosis. The management of cancer-associated abdominal vein thrombosis follows the general guidelines for the management of venous thromboembolism.

**Abstract:**

Cancer is associated with an increased risk of developing venous thromboembolism, due to its direct influence on the three pillars of Virchow’s triad (e.g., compression on the blood vessels by the tumour, blood vessels invasion, and cytokine release), together with the effect of exogenous factors (such as chemotherapy, radiotherapy, surgery). In cancer patients, the risk of thrombosis at unusual sites, such as splanchnic, ovarian and renal vein thrombosis, is also increased. Abdominal vein thromboses are frequently incidental findings on abdominal imaging performed as part of the diagnostic/staging workup or the follow-up care of malignancies. There is little evidence on the management of unusual site venous thromboembolism in cancer patients since there are only a few specific recommendations; thus, the management follows the general principles of the treatment of cancer-associated deep vein thrombosis and pulmonary embolism. This narrative review summarises the latest evidence on cancer-associated abdominal vein thrombosis, i.e., thrombosis of the splanchnic, ovarian and renal veins.

## 1. Introduction

Cancer is associated with an increased risk of developing venous thromboembolism (VTE), which can lead to increased morbidity, hospitalisation and mortality [1]. An Austrian nationwide epidemiological study highlighted that the risk of developing VTE was 15 times higher in cancer patients in comparison to patients without cancer. This risk is also increased in young patients, showing the relevant effect of cancer on an age category at intrinsically low risk of VTE. In addition, around 20% of all VTE patients had a cancer diagnosis, suggesting that 1 out of 5 VTE cases can be considered cancer-associated thrombosis (CAT) [2,3].

**Figure 1:**
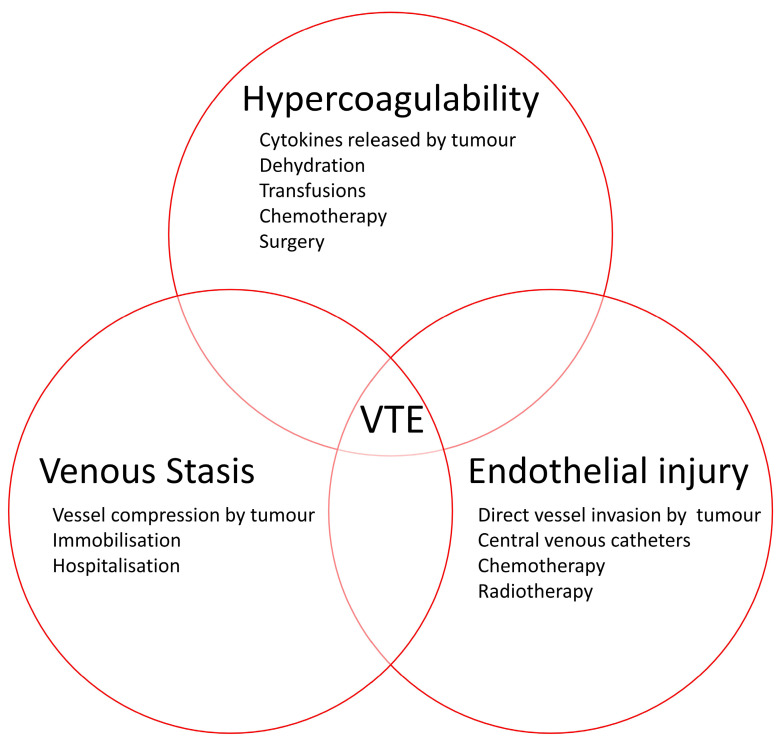
The Virchow’s triad in cancer patients. Legend: VTE = venous thromboembolism.

The description of the association between cancer and thrombosis dates back to the 19th century, when it was reported as Trousseau syndrome, taking its eponym from the French physician Armand Trousseau [4,5]. Nowadays the term Trousseau syndrome is more commonly referred to migratory thrombophlebitis as a sign of visceral cancer [6]. In the same century, the German pathologist Rudolph Virchow explained the pathogenesis of VTE, through the so-called Virchow’s triad (blood stasis, hypercoagulability and endothelial injury) which can also be applied to CAT (Figure 1) [7]. Venous stasis can be increased due to immobility, as well as tumour compression on blood vessels. Hypercoagulability can result from several biochemical factors released by the tumour (e.g., cytokines), but can also be due to the treatment itself (e.g., chemotherapy or recent surgery). Endothelial injury can occur due to direct effect of tumour invasion with damage to the endothelium, the presence of central venous catheters and the treatment of the malignancy (e.g., chemotherapy or radiotherapy) [8]. 

Thrombosis of the abdominal veins (i.e., thrombosis of the splanchnic, ovarian and renal veins) are considered unusual locations of VTE, because they are less common than deep vein thrombosis (DVT) and pulmonary embolism (PE). However, they are frequently detected in cancer patients. In a recent study enrolling 1702 patients with CAT, 167 (9.8%) had splanchnic and 49 (2.9%) had renal/ovarian vein thrombosis [9]. 

We performed a narrative review of the literature with the aim to summarise the latest evidence on cancer-associated abdominal vein thrombosis. 

## 2. Splanchnic Vein Thrombosis

### 2.1. Epidemiology

Splanchnic vein thrombosis (SVT) includes thrombosis of the portal vein, mesenteric veins, splenic vein and the Budd–Chiari syndrome (BCS). Among the different locations of SVT, the most common is portal vein thrombosis (PVT), with incidence rates of 1.73 and 3.78 cases per 100,000 persons per year in females and males, respectively, while the least common is BCS, with incidence rates of around 2 cases per million persons per year [10].

Cancer is one of the major risk factors for SVT. In the study by Thatipelli et al., the prevalence of solid cancer was higher in SVT patients compared to DVT patients (27% vs. 17%) [11]. The prevalence of solid cancer in incidentally detected SVT is even higher (35–64%) [12,13]. The most common tumours in cancer-associated SVT were hepatobiliary and pancreatic (57.6%), followed by gastrointestinal (25.8%) and genitourinary cancers (21.2%) [14]. In the study by Handa et al. which evaluated a US nationwide inpatients database, out of 32,324 patients who were admitted to hospital due to SVT between the years 2007 and 2017, 10% of them had a gastrointestinal malignancy, mainly liver (hepatocellular carcinoma) or pancreatic cancers [15]. In the study by Zanetto et al., the 1-year incidence of PVT was 24.4% in cirrhotic patients with hepatocellular carcinoma vs. 11.4% in cirrhotic patients without hepatocellular carcinoma [16].

There is a strong association between myeloproliferative neoplasms (MPN) and SVT. The mean prevalence of MPN was reported to be 40.9% in patients with BCS and 31.5% in patients with PVT [17], while the overall prevalence of SVT in MPN patients ranged from 5% to 13% [18].

Furthermore, SVT can be a marker of occult cancer. Approximately 8% of SVT patients will have a diagnosis of cancer in the 3 months following SVT, in particular liver cancer (absolute risk 3.5%), pancreatic cancer (absolute risk 1.5%) and MPN (absolute risk 0.7%) [19].

### 2.2. Clinical Presentation

Symptoms of SVT are non-specific: the most common is abdominal pain (48–55% of cases), followed by ascites (10–29%) and gastrointestinal bleeding (9–26%) [11,20]. Patients may also complain of other symptoms such as nausea, vomiting, anorexia, diarrhoea or constipation, and rarely fever. Furthermore, there might be some peculiar clinical scenarios based on the site of thrombosis. BCS can present with a triad of hepatomegaly, ascites, and abdominal pain. Mesenteric vein thrombosis (MVT) can present with intestinal infarction and acute abdominal pain in a third of patients. Chronic PVT is characterised by the presence of portal cavernoma and symptoms of portal hypertension [21]. Cancer-associated SVT is often detected in asymptomatic patients: an incidental diagnosis of SVT was reported in 44.7% of SVT patients with solid cancer [14].

### 2.3. Diagnosis

SVT diagnosis relies on imaging. D-dimer has high sensitivity (96%) but very low specificity (25%) for SVT [22]. Doppler Ultrasound (US) can be used as first-line imaging for PVT (sensitivity 89–93%, specificity 92–99%) and for BCS [23]. However, visualisation of the mesenteric veins can be difficult in obese patients and in the presence of meteorism. Thus, computed tomography (CT) angiography or magnetic resonance imaging (MRI) angiography are the preferred diagnostic modalities for MVT. CT and MRI can also be performed when US is inconclusive, and to evaluate thrombosis extension in PVT. 

In cancer patients, SVT is frequently an incidental finding discovered at follow-up imaging. In a study evaluating 2591 abdominal CT scans, the prevalence of incidentally detected abdominal vein thrombosis was 1.74% in the overall population, and 2.50% when considering only cancer patients [24].

### 2.4. Treatment

Anticoagulation is the mainstay treatment for SVT. It should be started early to prevent thrombus extension and increase the likelihood of recanalization. A guidance from the International Society on Thrombosis and Haemostasis (ISTH) divides the treatment of SVT based on the aetiopathogenesis. In non-malignant, non-cirrhotic SVT, it is suggested to use the direct oral anticoagulants (DOAC) as first-line treatment, and to consider low-molecular-weight heparin (LMWH) and vitamin K antagonists (VKA) if there are any contraindications to DOACs [25]. In patients with cancer-associated SVT, the recommended choices are LMWH or DOACs. However, a preference is given to LMWH in case of luminal gastrointestinal cancer or genitourinary cancer at high bleeding risk, and in patients receiving chemotherapy which might interact with the DOACs [25]. An indefinite treatment duration is recommended in SVT caused by persistent risk factors, with periodical reassessment of the risk of recurrent VTE and the risk of bleeding [26]. 

Recently, several studies focused on SVT in patients with solid cancer in general [14,27] or associated with gastrointestinal malignancies [28,29,30]. The proportion of patients receiving anticoagulant treatment in these studies was variable, ranging from ~18% to ~70%. In addition, the study by Valeriani et al. highlighted that solid cancer-associated SVT was less frequently anticoagulated than cancer-associated DVT and/or PE (68.9% vs. 99.2%) and that median treatment duration was shorter (6 vs. 11 months, respectively) [14]. Recent studies evaluating the treatment of solid cancer-associated SVT are detailed in Table S1. Of note, in cancer-associated SVT, mortality rates were relatively high (41–61%), while the rates of major bleeding (2.3–9.8%) and recurrent VTE (3.9–4.5%) were in line with a previous meta-analysis on this topic [31].

Incidentally detected SVT, which is not uncommon in cancer patients, should be treated like symptomatic SVT [25]. In fact, an individual patient data meta-analysis reported that incidental SVT had a higher risk of recurrent VTE and similar bleeding risk, compared to symptomatic SVT patients [32]. 

### 2.5. Prognosis

Overall, SVT carries a poor prognosis compared to the general population, with a 30-day mortality risk of 20.6% for SVT patients vs. 0.7% for a matched cohort of individuals without SVT. MVT was associated with the highest short-term mortality rate (63.1% at 30 days), due to the risk of bowel ischaemia, whereas PVT showed the highest mortality rate in the long-term (23.1% at 1 year, 27.2% at 5 years) [33]. Furthermore, SVT in general has lower survival rates than lower-limb DVT [11].

SVT patients with solid cancer had the highest mortality rate of 39.5 (95% CI, 31.1–50.1) per 100 patient years, compared to other subgroups of SVT patients [20]. However, the 1-year mortality rates were similar between cancer-associated SVT and cancer-associated VTE at typical locations (41.7% vs. 39.4%, respectively) [14]. In the study by Handa et al., SVT patients with gastric or hepatic cancer had the highest inpatient mortality rates (12.1% and 7.6%, respectively) [15]. Afzal et al. showed that patients with advanced pancreatic cancer who developed SVT had a two-fold increase in mortality rates (adjusted hazard ratio (aHR) 2.02; 95% CI, 1.65–2.47) [29].

Patients with MPN-associated SVT have better prognosis with a reported mortality rate of 3.4 (95% CI, 1.1–10.4) per 100 patient years [20]. Furthermore, the prognosis of MPN patients does not seem to be influenced by the presence of SVT [19].

The risk of recurrent VTE in cancer-associated SVT is relevant: 7.6 (95% CI, 4.3–13.3) per 100 patient years in patients with solid cancer, and 5.9 (95% CI, 2.5–14.3) per 100 patient years in patients with MPN [20]. However, the cumulative incidence of recurrent VTE in patients with solid cancer and SVT was not significantly different compared to patients with solid cancer and usual site VTE [14].

## 3. Ovarian Vein Thrombosis

### 3.1. Epidemiology

There are no precise estimates on the epidemiology of ovarian vein thrombosis (OVT) in the general oncological population. Most studies are focused on post-partum OVT and reported an incidence between 0.049% [34] and 0.18% [35]. 

There are three studies assessing the rates of post-operative OVT in patients with gynaecological malignancies after cancer surgery [36,37,38]. In the study by Yassa et al., out of 50 women with either cervical, ovarian or endometrial cancer, undergoing total abdominal hysterectomy with bilateral salpingo-oophorectomy, 40 patients (80%) had an incidental finding of unilateral OVT at follow-up CT scan. Mantha et al. enrolled 159 patients undergoing a routine CT scan 12 weeks post-operatively after debulking surgery for ovarian cancer and found incidental findings of OVT in 41 (25.8%) of them [38]. More recently, Takahashi et al. followed 417 patients after gynaecological cancer surgery including bilateral adnexectomy; 55 (13.2%) of them were diagnosed with incidental OVT within 6 months, with no significant difference found amongst patients with cervical, endometrial and ovarian cancers [36]. Studies evaluating the rates of incidentally detected OVT in patients undergoing gynaecological cancer surgery are summarised in Table S2.

Cancer is one of the most common causes of OVT. In studies enrolling different aetiologies of OVT, the most common risk factors were malignancy (11–60%), pregnancy/puerperium (9–61%) and abdominal surgery (6–70%) [39,40,41]. In the study by Lenz et al., 96 (44%) out of 219 patients with OVT had a malignancy and the prevalence of cancer was almost twice than in patients with lower limb DVT (21%). The most common cancer locations were genitourinary and gastrointestinal cancers [40]. Other studies have reported a lower rate of OVT associated with malignancy at 27%, whilst still being the most common cause of OVT [42]. OVT is associated with several other causes such as hormonal stimuli (e.g., pregnancy and post-partum), pelvic inflammatory disease, surgery, and hospitalisation [39,40,43].

While pregnancy-related OVT more commonly involves the right ovarian vein, due to the dextrorotation and compression from the gravid uterus [44], studies including mixed aetiologies reported similar involvement of both ovarian veins [39,40,42]. Nonetheless, in the study by Yassa et al. enrolling women after gynaecological cancer surgery, the right ovarian vein was involved in 75% of OVT cases. All these patients had complete occlusion of the vein on the CT scan; however, none presented with any signs or symptoms, and none were treated with anticoagulation [37]. OVT extension into the renal vein or the inferior vena cava is commonly reported, up to 25% and 31% of cases, respectively [45].

### 3.2. Clinical Presentation

The clinical presentation of OVT typically includes abdominal pain, usually in the lower quadrant ipsilateral to the thrombosed vein, with rebound tenderness and guarding. The pain can radiate to the pelvis and to the back. Post-partum OVT can present with fever within 96 h of delivery and generally unresponsive to antibiotics, which led to its description as septic pelvic thrombophlebitis [43,46]. A lower abdominal mass can be palpated in many cases, which corresponds to the thrombosed vein [35]. Other symptoms include diaphoresis and tachypnoea [43]. 

Oncological patients with OVT are commonly asymptomatic. Studies performing postoperative imaging after gynaecological cancer surgery reported that OVT was frequently an incidental finding, since all patients who developed OVT had no symptoms [36,37,38]. 

### 3.3. Diagnosis

Doppler US can diagnose OVT by showing the reduction or lack of blood flow in the ovarian veins [43]. US is limited by abdominal meteorism and patient’s body habitus, which can impair the visualisation of the ovarian veins. However, postpartum US scans performed in patients with no previous history of thromboembolism, after vaginal delivery, showed that, in 78.6% of women, both ovarian veins could be visualised [47]. CT and MRI are the recommended imaging tests, since they can more easily visualise the OVT [48]. Based on available studies, the reported ranges of sensitivity and specificity for US were 50–100% and 41–99%, for CT scan 77–100% and 62–99%, and for MRI scan 92–100% and ~100%, respectively [49].

The Society of Obstetricians and Gynaecologists of Canada (SOGC) suggests that a pelvic US can be performed as a first line investigation. However, CT or MRI (preferably with angiography) should be considered as the definitive imaging modalities to confirm or rule out the presence of OVT, especially when the US is inconclusive [50].

### 3.4. Treatment

The SOGC Guidelines addressing VTE during pregnancy and post-partum recommended parenteral broad-spectrum antibiotics for at least 48 h after defervescence, with a longer duration if the patient has a complicated infection. OVT should also be managed with therapeutic anticoagulation for 1–3 months [50]. The British Committee for Standards in Haematology (BCSH) Guidelines for venous thrombosis at unusual sites provided recommendations for the management of OVT based on the causative factor. Postpartum OVT should be treated with typical anticoagulation for 3–6 months, in line with the management of VTE at usual locations. However, a significant proportion of OVT diagnosis is due to incidental findings, typically during imaging for gynaecological surgery. They stated that incidental OVT findings in cancer patients do not require anticoagulant treatment, except for patients with inferior vena cava extension or PE [48]. 

This management has been seen in several retrospective cohort studies in which patients were treated at the physician’s discretion. Lenz et al. reported that OVT was less frequently treated with anticoagulation in comparison to leg DVT (54% vs. 98%, respectively, *p* < 0.001) and that, in turn, cancer-associated OVT was less frequently anticoagulated than non-cancer-associated OVT (41% vs. 64%, respectively, *p* < 0.01) [40]. Mantha et al. showed that among 41 patients with ovarian cancer and OVT, only 5 (12.2%) of them were anticoagulated and the drug of choice was LMWH [38]. Takahashi et al. reported that among 55 women with OVT and gynaecological malignancies after adnexectomy, only 6 (10.9%) patients were anticoagulated (3 received VKA and 3 DOACs). Although there was no significant difference in OVT resolution between treated and untreated patients (83.3% vs. 75.5%, *p* = 0.32), 4 (8.2%) out of 49 untreated patients had OVT progression, which did not extend into the renal vein or the inferior vena cava [36]. The study by Assal et al. enrolled 223 OVT patients, of whom 60.1% had a diagnosis of cancer. Anticoagulated patients had lower VTE recurrence rates compared to untreated patients (5.9% vs. 9.9%, *p* = 0.59); however, the difference was not statistically significant, probably due to the low number of treated patients (~9%) [39].

### 3.5. Prognosis

In a case–control study comparing OVT patients with age- and gender-matched DVT patients, VTE recurrence rates were similar in the two groups (2.3 vs. 1.8 per 100 patient years, respectively), despite OVT patients being less frequently anticoagulated. Furthermore, in OVT patients, the presence of cancer did not significantly increase the risk of VTE recurrence. Mortality rates were also similar between OVT and DVT; however, the presence of cancer was associated with worse survival rates for patients with OVT compared to non-cancer associated OVT [40].

In the study by Mantha et al. assessing the incidence of OVT after surgical resection of ovarian cancer, overall survival rates were not significantly affected by the development of OVT after surgery (95.1% in patients with OVT vs. 93.2% in patients without OVT at 1-year follow-up). In addition, despite only a minority of OVT patients being anticoagulated, there was no significant increased risk of VTE between patients with and without OVT (17.1% vs. 15.3% at 1-year follow-up) [38]. 

## 4. Renal Vein Thrombosis 

### 4.1. Epidemiology

There are no precise estimates regarding the epidemiology of renal vein thrombosis (RVT) in cancer patients. RVT is a rare medical condition, with an incidence rate of ≤0.1 cases per 100,000 persons per year in the general population [51]. It more commonly arises in neonates, in whom the annual incidence rate was reported to be 2.6 per 100,000 live births [52]. RVT is frequently associated with nephrotic syndrome and is detected in 5–62% of patients with this condition [53]. Males are more commonly affected than females due to the greater occurrence of membranous nephropathy, which is a predisposing factor for RVT [54]. The left renal vein is more prone to thrombosis due to its longer course and complex venous system, as opposed to the right renal vein [55]. RVT extension into the inferior vena cava is common, being reported in 43–46% of patients [56,57].

Cancer is one of the most common causes of RVT in adults. In the cohort study by Rottenstreich et al. [57], 19 out of 39 patients with RVT (48.7%) had active cancer at the time of RVT diagnosis. In the study by Wysokinski et al., 143 out of 218 patients with RVT (66.2%) had an underlying malignancy, and the prevalence of cancer was almost 3 times higher than in patients with lower limb DVT (21.6%) [56]. In both studies, the most common tumour was renal cell carcinoma [56,57], which in general accounts for 90% of all renal malignancies [58]. In 10% of patients with renal cell carcinoma, the tumour grows along the renal vein (tumour thrombus) and can extend into the inferior vena cava [58,59]. Cancers less frequently associated with RVT include other urinary malignancies (such as Willms tumour and urothelial carcinoma of the renal pelvis), gynaecological and gastrointestinal tumours, and more rarely adrenocortical carcinoma and hepatocellular carcinoma [60,61,62].

### 4.2. Clinical Presentation

The clinical manifestations of RVT differ based on the acute or chronic presentation of thrombosis, since the rate of venous occlusion and the formation of collateral venous pathways affect its clinical manifestations [63]. Acute RVT is accompanied by symptoms of flank pain and tenderness, gross haematuria, nausea, and vomiting [53]. Acute RVT is common in neonates with dehydration, prematurity and perinatal asphyxia [64]. Bilateral RVT can lead to acute renal failure [48]. Chronic RVT can be asymptomatic or paucisymptomatic, with the subtle presentation of peripheral oedema, but may lead to kidney impairment [53]. However, there is a higher prevalence of acute (64%) over chronic RVT [57]. Cancer patients with RVT are generally older than non-cancer patients (median age 66 vs. 28 years, respectively) and more commonly present with chronic (36.8% vs. 5.0%) or asymptomatic (21.1% vs. 10.0%) RVT [57].

### 4.3. Diagnosis

Renal venography used to be the gold standard; however, it is rarely used nowadays due to the availability of alternative diagnostic methods which are less invasive [48]. 

US can be used as the initial imaging modality [53]. It shows enlarged kidneys with increased echogenicity, and lack of blood flow in the renal vein. However, sensitivity and specificity for the detection of RVT have been reported to be 85% and 56%, respectively, compared to venography [65].

CT angiography is the imaging of choice, with almost 100% sensitivity and specificity compared to digital subtraction angiography [63,66]. MRI angiography can be used as an alternative to CT scan with reported sensitivity of 94.1% and specificity of 100%, compared to CT venography [67].

### 4.4. Treatment

Therapy for RVT should be aimed at preventing thrombus progression and embolization, and at preserving renal function [68]. Anticoagulation is the main treatment for RVT; however, lacking specific recommendations, the management usually follows the same principles as the treatment of DVT and PE [48]. This uncertainty is also represented by the fact that the proportion of anticoagulated RVT patients ranged from 52.3% to 71.8% in different studies [56,57].

In general, treatment is started with unfractionated heparin (UFH) or LMWH and can be continued with oral anticoagulants. In the study by Wysokinski et al., out of 143 patients with CAT, 60 (42.0%) received heparin and 24 (16.8%) received warfarin [56]. In a small case series of 8 RVT patients treated with DOAC, 6 of them (75%) had active malignancy [69].

In patients with cancer-associated RVT, renal function impairment is a common occurrence and thus the dose of certain anticoagulants (such as LMWH and DOACs) might need adjustment [70,71]. 

Duration of anticoagulant therapy differed in the published studies. In the cohort by Rottenstreich et al., patients with active cancer received anticoagulation for 3–12 months, while in the cohort by Wysokinski et al. approximately half of the patients were treated with VKAs for a duration up to one year, and the other half were treated lifelong.

Thrombectomy and/or thrombolysis can be considered only in patients with acute bilateral RVT and acute renal failure, if no response to anticoagulant treatment [63]. 

### 4.5. Prognosis

The prognosis of RVT depends on the underlying risk factors and the degree of renal function alteration. In general, RVT survival is lower compared to lower-limb DVT. Wysokinski et al. reported that the overall mortality rate in RVT patients was 18.0 per 100 patient-years, and that cancer-associated RVT was associated with increased mortality risk compared to nephrotic syndrome-associated RVT [56]. In the study carried out by Rottenstreich et al., 28% of patients died, However, all these patients had active cancer and all deaths were not related to RVT. The mortality rate was significantly higher in cancer patients compared to non-cancer patients, thus confirming that the prognosis of RVT is highly dependent on the presence of active malignancy [57].

In general, the risk of recurrent VTE in RVT patients is significantly lower than the risk of recurrent VTE in patients with lower limbs DVT [56], and most recurrences consist of DVT (recurrent RVT is rare). In the study carried out by Wysokinski et al., the risk of recurrent VTE was 1.0 per 100 patient years. Despite most of the events (5 out of 8) occurring in cancer patients, the risk of recurrence was not significantly affected by the presence of malignancy [56].

## 5. Guidelines for Cancer-Associated Venous Thromboembolism

**Figure 2:**
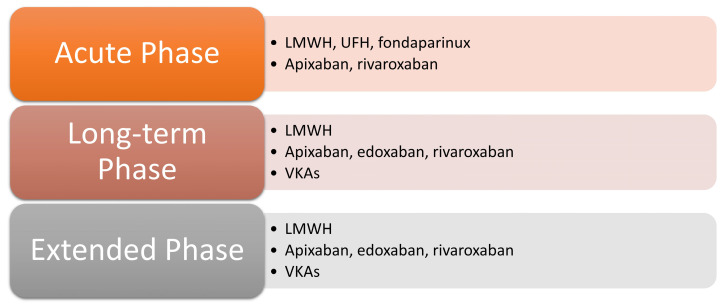
The anticoagulant treatment of cancer-associated thrombosis. Legend: LMWH = low-molecular-weight heparin; UFH = unfractionated heparin; VKAs = vitamin K antagonists.

The anticoagulant treatment of cancer-associated abdominal vein thrombosis follows the general principles of the treatment of cancer-associated DVT and PE, since there are only a few specific recommendations. The treatment of CAT is generally divided into three phases: acute phase (5–10 days after diagnosis); long-term phase (up to 6 months); extended phase (after 6 months) (Figure 2) [72].

For the acute phase possible anticoagulant options include LMWH, UFH, fondaparinux, apixaban or rivaroxaban [72]. LMWH showed a trend towards lower mortality rate (risk ratio (RR) 0.66; 95% CI, 0.40–1.10) and lower recurrent VTE (RR 0.69; 95% CI, 0.27–1.76) compared to UFH. Fondaparinux showed a trend towards higher mortality rate (RR 1.25; 95% CI, 0.86–1.81) and higher minor bleeding events (RR 1.53; 95% CI, 0.88–2.66) compared to LMWH [73]. Thus, LMWH is the preferred choice compared to UFH or fondaparinux. However, UFH may be considered in patients with severe renal impairment and creatinine clearance <30 mL/min, while fondaparinux may be considered in patients with previous heparin-induced thrombocytopenia [72]. 

For the long-term phase, possible anticoagulant options include LMWH, apixaban, edoxaban, rivaroxaban or VKAs [72]. LWMH has been the cornerstone of the treatment for CAT, since it showed significantly lower recurrent VTE (RR 0.59; 95% CI, 0.44–0.80) compared to VKAs, without increasing the risk of major bleeding [73]. VKAs might also have drug–drug interactions with chemotherapy drugs [71]. Recently, multiple randomized controlled trials have shown that the DOACs (the factor Xa inhibitors apixaban, edoxaban, and rivaroxaban) are an adequate alternative to LMWH, with an added ease of use for patients due to their oral administration at fixed doses [74]. Of note, while apixaban and rivaroxaban can be started as a single drug approach using higher doses for the initial weeks, edoxaban requires some days of LMWH as induction. The DOACs showed a trend towards lower recurrent VTE (RR 0.63; 95% CI, 0.34–1.15) compared to VKAs. They also showed significantly lower recurrent VTE (RR 0.63; 95% CI, 0.45–0.88), significantly higher minor bleeding events (RR 1.58; 95% CI, 1.15–2.16) and a trend towards higher major bleeding events (RR 1.20; 95% CI, 0.83–1.73) compared to LMWH [73]. Thus, LMWH and direct factor Xa inhibitors are preferred over VKAs. However, among the different direct factor Xa inhibitors, edoxaban and rivaroxaban were associated with increased risk of gastrointestinal major bleeding, while apixaban was associated with the lowest bleeding risk [74]. Thus, LMWH is preferable over DOACs in patients with luminal gastrointestinal or urothelial cancer, high risk of gastrointestinal bleeding (e.g., active gastroduodenal ulcer), or receiving chemotherapy interfering with DOACs (e.g., strong inducers or inhibitors of CYP34A or P-glycoprotein) [72]. 

For the extended phase, possible anticoagulant options include LMWH, apixaban, edoxaban, rivaroxaban or VKAs [72]. The duration of anticoagulation is typically extended beyond 6 months in patients with active cancer when the risk of recurrent VTE outweighs the risk of bleeding. 

Furthermore, the recent clinical practice guidelines of the European Society for Medical Oncology (ESMO) recommended treatment of incidentally detected CAT in the same way as symptomatic CAT [72]. Previously, the 2012 guidelines of the British Committee for Standards in Haematology (BCSH) reported that incidentally detected OVT in cancer patients after hysterectomy does not necessarily require anticoagulation, unless there is extension into the inferior vena cava or associated PE [48]. The 2023 Guidelines of the American Society of Clinical Oncology (ASCO) suggested to consider anticoagulation for incidentally detected splanchnic or visceral vein thrombosis on a case-by-case basis [75]. Finally, the 2020 ISTH guidance specifically addressed the treatment of cancer-associated SVT. The authors recommended LMWH or DOAC, with a preference for LMWH if luminal gastrointestinal or genitourinary cancer had a high bleeding risk, or chemotherapy interacting with DOACs [25].

## 6. Conclusions

Despite being unusual locations of VTE, thromboses of the splanchnic, ovarian and renal veins are not uncommon in cancer patients. Cancer is one of the main risk factors, being 2–3 times more common in abdominal vein thrombosis than in lower-limb DVT. In fact, they are often detected as incidental findings at abdominal imaging performed as part of the diagnostic workup or the follow-up care of malignancies. Furthermore, among the different aetiopathogenesis of thrombosis, cancer-associated SVT, OVT and RVT have higher mortality rates compared to their non-cancer-associated counterparts. A summary of the characteristics of cancer-associated abdominal vein thrombosis is reported in Table 1.

There is limited literature regarding cancer-associated abdominal vein thrombosis. The rarity of these abdominal thrombosis resulted in a small number of studies available for each of these locations. Additionally, most available studies have an observational design, a small sample size, a short follow-up and heterogeneous outcome definitions. There is also limited evidence on the management of cancer-associated abdominal vein thrombosis, since only a minority of patients enrolled in the existing studies received anticoagulant treatment. Furthermore, with a few exceptions, current guidelines do not specifically address these thromboses. As a consequence, the management of cancer-associated abdominal vein thrombosis in clinical practice follows the management of cancer-associated VTE in more common locations. Future research should focus on performing large collaborative studies, preferably with a prospective design, using standardized outcome definitions [76].

## Figures and Tables

**Table 1:** Summary characteristics of cancer-associated abdominal vein thrombosis.

Thrombosis	Epidemiology	Clinical Presentation	Diagnosis	Treatment	Prognosis
Splanchnic vein thrombosis	Prevalence of solid cancer in SVT overall ~27%, in incidentally detected SVT ~35–64% (higher than usual site VTE) Most common tumors: hepatobiliary and pancreatic (~58%), gastrointestinal (~26%), genitourinary (~21%) Prevalence of MPN in BCS ~41%, in PVT ~32% Within 3 months after SVT: risk of liver cancer 3.5%, pancreatic cancer 1.5%, MPN 0.7%	Abdominal pain, ascites, gastrointestinal bleeding, nausea, vomiting, anorexia, diarrhoea, constipation, fever Hepatomegaly, ascites, abdominal pain (BCS) Intestinal infarction (MVT) Portal cavernoma, portal hypertension (chronic PVT) Asymptomatic incidentally detected SVT in ~45% of solid cancer patients	Doppler US: first line for PVT and BCS CT or MRI angiography: better visualization of mesenteric veins and thrombosis extension	Start anticoagulation early For cancer-associated SVT: possible options are LMWH or DOAC. Preference for LMWH if luminal gastrointestinal cancer, genitourinary cancer at high bleeding risk, chemotherapy interfering with DOAC Indefinite treatment duration, with periodical reassessment of thrombotic and bleeding risk Incidentally detected SVT should be treated like symptomatic SVT	Mortality rates in solid cancer-associated SVT 39.5 per 100 pt-y, MPN-associated SVT 3.4 per 100 pt-y Recurrent VTE in solid cancer-associated SVT 7.6 per 100 pt-y, MPN-associated SVT 5.9 per 100 pt-y
Ovarian vein thrombosis	Prevalence of cancer in OVT ~44% (higher than usual site VTE) Most common tumors: genitourinary (~50%), gastrointestinal (~29%) Rates of incidentally detected OVT post-gynecological cancer surgery 13-80%	Abdominal pain and tenderness, fever, abdominal mass Asymptomatic incidentally detected OVT is common in cancer patients	Doppler US: first line CT or MRI angiography: better visualization of the ovarian veins	Broad spectrum antibiotics until 48 h after defervescence Anticoagulation for at least 3 months Treatment of incidentally detected OVT in cancer patients is still debated	Mortality rates in cancer-associated OVT: ~5% at 1 year follow-up Recurrent VTE in cancer-associated OVT: ~17% at 1 year follow-up
Renal vein thrombosis	Prevalence of cancer in RVT ~48–66% (higher than usual site VTE) Most common tumor: renal cell carcinoma (~58–78%)	Flank pain and tenderness, gross haematuria, nausea, vomiting (acute RVT) Asymptomatic or pauci-symptomatic (chronic RVT) Asymptomatic incidentally detected RVT is common in cancer patients	Doppler US: first line CT or MRI angiography: better visualization of the renal veins	No specific recommendations for RVT Some anticoagulants might need dose adjustment based on renal function	Mortality rates in cancer-associated RVT: ~47% at 19 months follow-up Recurrent VTE in cancer-associated RVT: ~3% at 42 months follow-up

Legend: BCS = Budd-Chiari syndrome, CT = computed tomography, DOAC = direct oral anticoagulants, LMWH = low molecular weight heparin, MPN = myeloproliferative neoplasms, MRI = magnetic resonance imaging, MVT = mesenteric vein thrombosis, OVT = ovarian vein thrombosis, pt-y = patient-years, PVT = portal vein thrombosis, RVT = renal vein thrombosis, SVT = splanchnic vein thrombosis, US = ultrasound, VTE = venous thromboembolism.

## Supplementary Materials

The following are available online at https://www.mdpi.com/article/10.3390/cancers15215293/s1, Table S1: Recent studies evaluating the anticoagulant treatment of solid-cancer-associated splanchnic vein thrombosis; Table S2: Studies evaluating the epidemiology of ovarian vein thrombosis in cancer patients.
