# Supplementary material for: Cancer-Associated Abdominal Vein Thrombosis
Source: Cancers (Basel). 2023 Nov 4;15(21):5293. doi: 10.3390/cancers15215293 (PMC10649304; doi:10.3390/cancers15215293)
Supplement: Supplementary file 1 [file cancers-15-05293-s001.zip › cancers-2673405-supplementary.pdf]

Supplementary Table S1. Recent studies evaluating the anticoagulant treatment of solid-cancer-associated splanchnic vein thrombosis.

Author (year)	*Study design*	*N. patients with SVT and cancer*	*Patient characteristics*	*Management*	*Recurrent VTE*	*Major bleeding*	*Mortality*
**Regnault H. et al, 2018 [28]**	Retrospective	118	Patients with PVT and digestive cancers (other than hepatocellular carcinoma)	82/118 (69.5%) pts received anticoagulant treatment (not further specified)	NR	22/118 (18.6%) pts had gastrointestinal bleeding (severity not specified):<br>• 13/82 (15.9%) anticoagulated pts<br>• 9/36 (25.0%) non-anticoagulated pts	70/118 (59.3%) pts died (not separately reported for treated vs. non-treated)
**Sule A.A. et al, 2018 [27]**	Retrospective	13	Patients with PVT and malignancy	6/13 (46.2%) pts received anticoagulant treatment:<br>• LMWH n=5<br>• VKA n=1<br>• Mean duration 75 days	No events	Not separately reported for cancer-patients	8/13 (61.5%) pts died:<br>• 4/6 (66.7%) anticoagulated pts<br>• 4/7 (57.1%) not anticoagulated
**Afzal A. et al, 2020 [29]**	Retrospective	122	Patients with SVT and advanced pancreatic cancer	51/122 (41.8%) pts received anticoagulant treatment:<br>• LMWH n=37<br>• VKA n=11<br>• Fondaparinux n=3	NR	12/122 (9.8%) pts had major bleeding:<br>• 7/51 (13.7%) anticoagulated pts<br>• 5/71 (7.0%) non-anticoagulated pts	SVT doubled 1-year mortality (compared to pts without SVT). No significant difference between patients who did vs. did not receive anticoagulation.
**Valeriani E. et al, 2021 [14]**	Prospective	132	Patients with SVT and solid cancer	91/132 (68.9%) pts received anticoagulant treatment:<br>• Parenteral n=61<br>• VKA n=30<br>• Median duration 6 m	6/132 (4.5%) pts had recurrent VTE:<br>• 4/91 (4.4%) anticoagulated pts<br>• 2/41 (4.9%) non-anticoagulated pts	3/132 (2.3%) pts had major bleeding:<br>• 3/91(3.3%) anticoagulated pts<br>• 0/41 (0%) non-anticoagulated pts	55/132 (41.7%) pts died (not separately reported for treated vs. non-treated)
**Kang M. et al, 2022 [30]**	Prospective	51	Patients with SVT and gastrointestinal cancer	9/51 (17.6%) pts received anticoagulant treatment:<br>• LMWH n=6<br>• VKA n=1<br>• DOAC n=2<br>• Median duration 4.7 m	2/51 (3.9%) pts had recurrent VTE (not separately reported for treated vs. non-treated)	2/9 (22.2%) anticoagulated pts had major bleeding (NR for the non-anticoagulated group)	23/51 (45.1%) pts died (not separately reported for treated vs. non-treated)

Legend: DOAC = direct oral anticoagulants, LMWH = low molecular weight heparin, m = months, NR = not reported, pts = patients, PVT = portal vein thrombosis, SVT = splanchnic vein thrombosis, VKA = vitamin K antagonist, VTE = venous thromboembolism.

Supplementary Table S2. Studies evaluating the epidemiology of ovarian vein thrombosis in cancer patients.

Author (year)	*N. patients enrolled*	*Population characteristics*	*Years of enrolment*	*Anticoagulant prophylaxis*	*Imaging and timing*	*N. patients with OVT*	*OVT symptoms*	*OVT treatment*	*OVT outcome*
**Yassa N. et al, 1999 [37]**	50	Pts who underwent total abdominal hysterectomy and bilateral salpingo-oophorectomy with retroperitoneal lymph node dissection, due to:<br>• cervical ca (n=15)<br>• endometrial ca (n=5)<br>• ovarian ca (n=30)	1992-1996	NR	Contrast-enhanced CT scan every 3 months, up to 2 years after surgery	40/50 (80%)	All asymptomatic	None	• Thrombosis unchanged at 3-24 months in 20 out of 20 OVT pts (100%) with available CT scan<br>• None of OVT pts developed signs or symptoms of PE
**Mantha S. et al, 2015 [38]**	159	Pts who underwent debulking surgery, due to:<br>• ovarian ca (n=159)	2001-2010	NR	Contrast-enhanced CT scan within 12 weeks after surgery	41/159 (25.8%)	All asymptomatic	Only 5 patients were anticoagulated (LMWH n=5)	• VTE at 1 year: 17.1% in OVT pts, vs. 15.3% in non-OVT pts (p=0.78)<br>• Survival at 1 year: 95.1% in OVT pts, vs. 93.2% in non-OVT pts (p=0.84)
**Takahashi Y. et al, 2021 [36]**	417	Pts who underwent bilateral adnexectomy, due to:<br>• cervical ca (n=60)<br>• endometrial ca (n=188)<br>• ovarian ca (n=169)	2013-2017	387 (92.8%)	Contrast-enhanced CT scan within 6 months after surgery	55/417 (13.2%)	All asymptomatic	Only 6 patients were anticoagulated (VKA n=3, DOAC n=3)	• VTE at 1 year: 1.8% in OVT pts, vs. 2.8% in non-OVT pts (p=1.0)<br>• Survival at 3 years: 92.6% in OVT pts, vs. 83.3% in non-OVT pts (p=0.17)

Legend: ca = cancer; CT = computed tomography; DOAC = direct oral anticoagulants; LMWH = low molecular weight heparin; NR= not reported; OVT = ovarian vein thrombosis; PE = pulmonary embolism; pts = patients; VKA = vitamin K antagonists.
